# Supplementary figures and images for: Trends in injury hospitalisations due to contact with snakes in Australia, 2002 to 2020: A registry data analysis for the Australian Venomous Injuries Project (AVIP)
Source: PLoS Negl Trop Dis. 2025 Dec 15;19(12):e0013763. doi: 10.1371/journal.pntd.0013763 (PMC12704875; doi:10.1371/journal.pntd.0013763)

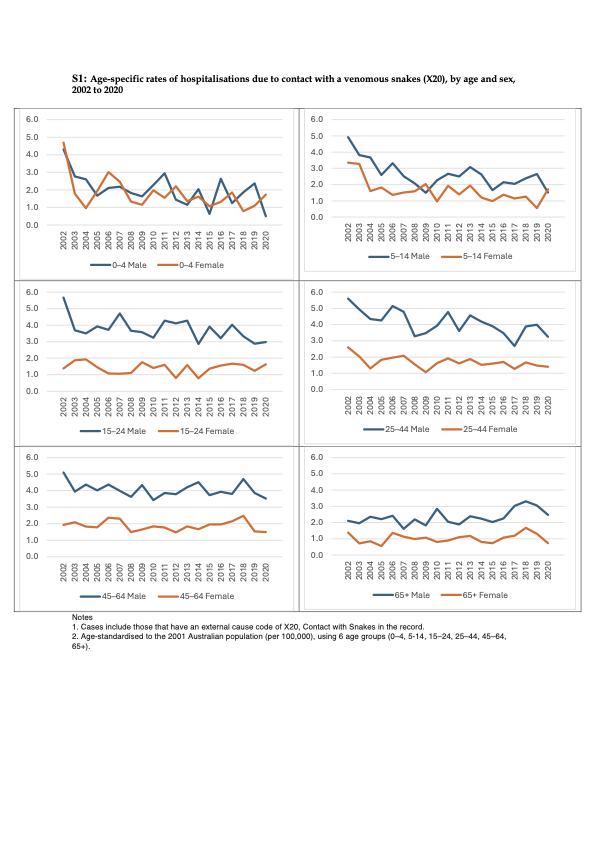

Supplement: S1 Fig — (TIFF) [file pntd.0013763.s001.tiff]

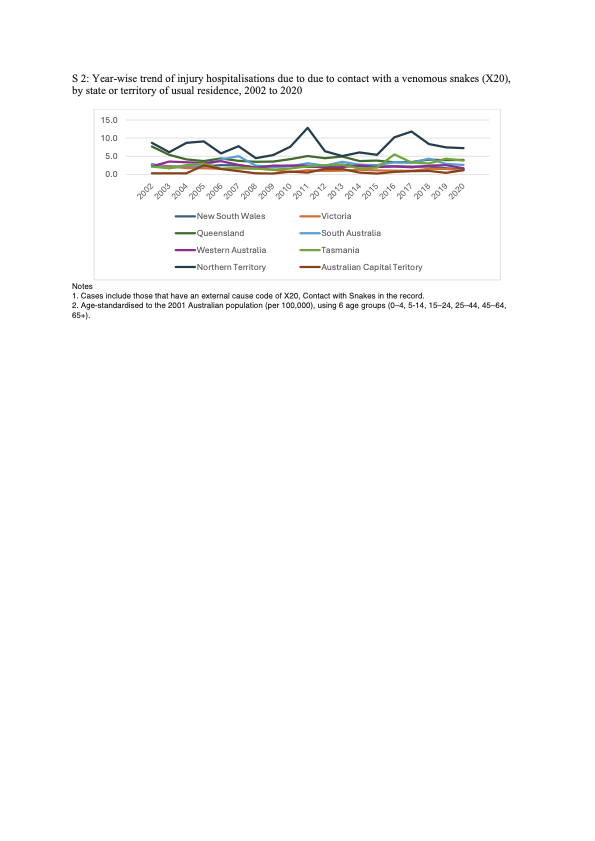

Supplement: S2 Fig — (TIFF) [file pntd.0013763.s002.tiff]

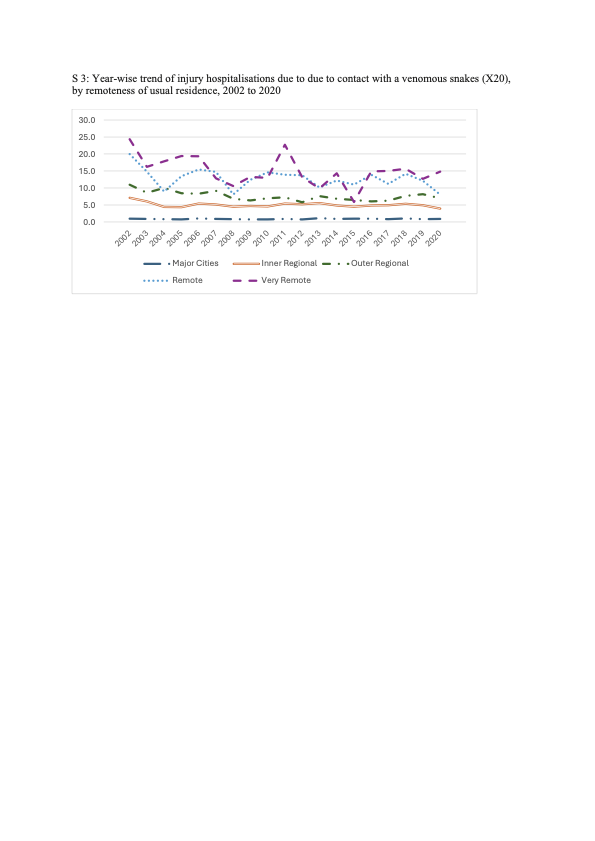

Supplement: S3 Fig — (TIFF) [file pntd.0013763.s003.tiff]

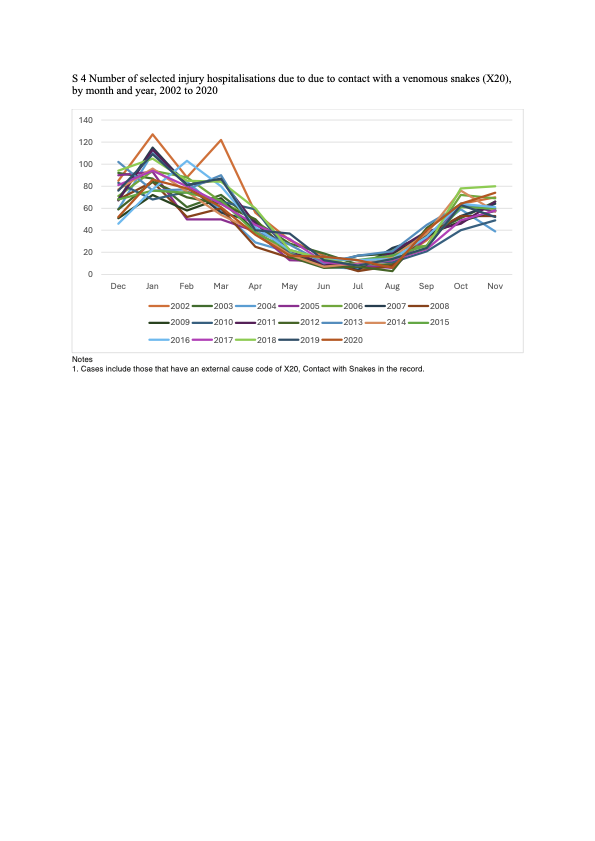

Supplement: S4 Fig — (TIFF) [file pntd.0013763.s004.tiff]

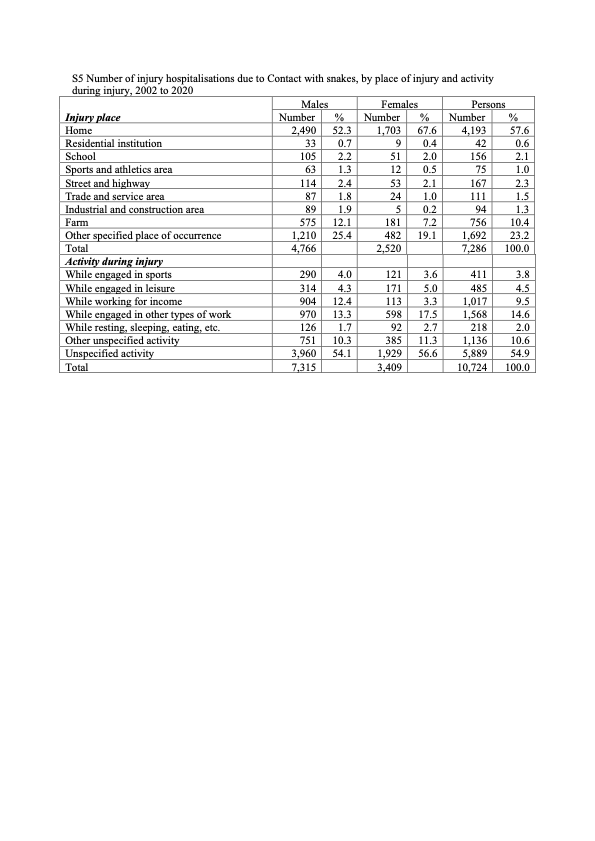

Supplement: S5 Table — (TIFF) [file pntd.0013763.s005.tiff]

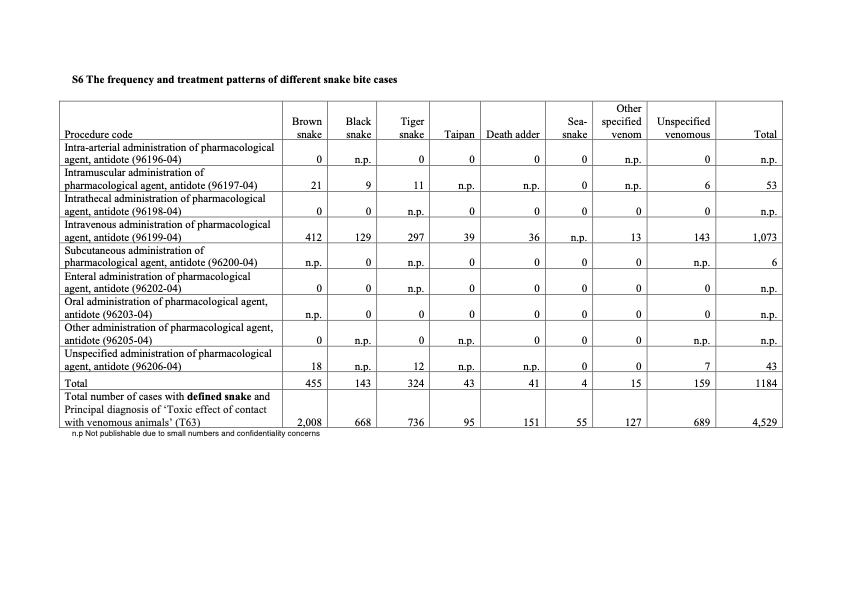

Supplement: S6 Table — (TIFF) [file pntd.0013763.s006.tiff]
