# Supplementary material for: Trends in injury hospitalisations due to contact with snakes in Australia, 2002 to 2020: A registry data analysis for the Australian Venomous Injuries Project (AVIP)
Source: PLoS Negl Trop Dis. 2025 Dec 15;19(12):e0013763. doi: 10.1371/journal.pntd.0013763 (PMC12704875; doi:10.1371/journal.pntd.0013763)
Supplement: Attachment — Submitted filename: Response to reviewers commentS_AAfroz.docx [file pntd.0013763.s008.docx]

To:

Wayne Hodgson

Academic Editor

PLOS Neglected Tropical Diseases

27 October 2025

Dear Wayne Hodgson,

Re: (PNTD-D-25-00380) - "Trends in injury hospitalisations due to contact with snakes in Australia, 2002 to 2020: A registry data analysis for the Australian Venomous Injuries Project (AVIP)"

Thank you for taking the time to review and provide feedback on our manuscript. We appreciate having an opportunity to improve our work.

Our responses to the Reviewer comments are provided below and changes in the manuscript are tracked with major changes highlighted where possible to make them easier to find.

We look forward to your response.

All the best,

Afsana Afroz

**Reviewers' Comments:**

**Reviewer's Responses to Questions**

**Key Review Criteria Required for Acceptance?**

**As you describe the new analyses required for acceptance, please consider the following:

Methods:

-Are the objectives of the study clearly articulated with a clear testable hypothesis stated?**

**-Is the study design appropriate to address the stated objectives?**

**-Is the population clearly described and appropriate for the hypothesis being tested?**

**-Is the sample size sufficient to ensure adequate power to address the hypothesis being tested?
-Were correct statistical analysis used to support conclusions?**

**-Are there concerns about ethical or regulatory requirements being met?**

**Reviewer #1: The aims are described, although these are rather unattainable aims, which the paper ultimately doesn't address. The study design is limited by the source of the data which is routinely collected data from hospital, which can only provide general information on demographics, geographical differences and limited treatment data. The statistical analysis is described.**

The aims and objectives of the current study have been clarified in line with the above comment. Please see below for the amended text.

*“The Australian Venomous Injuries Project (AVIP) has previously investigated patterns of venomous injury between 2000-2013 using national incidence data from the Australian Institute of Health and Welfare (AIHW)(7). Here, we have extended this earlier work with an investigation of hospitalisation data due to snakebite collected between 2002 to 2020. The objectives of our analyses are to provide insights into the trends and implications of injury hospitalisations due to contact with venomous snakes in Australia. This registry data analysis aims to describe the patterns or trends of hospitalisations over the study period, examining variations across different demographics, geographic locations, and types of venomous snakes. It is hoped that this investigation will provide additional insights into the burden of snakebite envenoming across Australia and identify emerging trends to assist healthcare providers, policymakers, and public health officials in their efforts to mitigate the impact of snakebite on the Australian population.”*

**Reviewer #2: -Are the objectives of the study clearly articulated with a clear testable hypothesis stated? Objectives are not clearly mentioned**

We thank the reviewer for their comment. The objectives have now been clarified as noted above.

**-Is the study design appropriate to address the stated objectives? To certain extend**

**-Is the population clearly described and appropriate for the hypothesis being tested? Yes**

**-Is the sample size sufficient to ensure adequate power to address the hypothesis being**

**tested? Not applicable**

**-Were correct statistical analysis used to support conclusions? Yes**

**-Are there concerns about ethical or regulatory requirements being met? No**

We thank the reviewer for their responses.

**Reviewer #3: The Aim of the study is not clear and should be explicitly stated
The methods are too brief and very confusing to an international reader. Methods should be more detailed and divided into subtopics such as Data Source, Data Authenticity, Data Extraction, Definitions, Data analysis.**

The aim of the study has been clarified as noted above and the methods re-organised and clarified in line with the reviewer comments.

**Information such as the identification of snake species (E.g. Brown snake etc.) should be stated in detail, how this identification is made in hospital settings and included in the database (E.g. specimen identification, venom detection kit etc?)**

The following text has been added to the discussion to highlight how snake type determinations are made in the clinical setting.

*‘It is noted that the recorded identification of snake species in many of these cases are unlikely to be definitive, instead relying on some combination of clinical presentation, descriptions or photos provided by patients, knowledge of venomous regional species, consultation with state-based Poisons Information Centre, and clinical toxinologists, and the use of snake venom detection kits (SVDKs), when and where available.’*

**Methods should include definitions. Remove all definitions in the results and include them in the methods. E.g. “Envenoming was considered to have occurred when the principal diagnosis was listed as toxic effect from contact with venomous animals (T63.0).” currently in the results should be moved to methods.**

We greatly appreciate your feedback. The method section has been revised as suggested.

**Results:

-Does the analysis presented match the analysis plan?**

**-Are the results clearly and completely presented?**

**-Are the figures (Tables, Images) of sufficient quality for clarity?**

**Reviewer #1: The analysis is presented as per the Methods, but it is rather complicated in multiple tables and quite difficult to understand.**

We greatly appreciate your feedback and have amended the methods to increase

clarity as noted above.

**Reviewer #2: Does the analysis presented match the analysis plan? Yes**

**-Are the results clearly and completely presented? Yes, see specific comments below**

**-Are the figures (Tables, Images) of sufficient quality for clarity? Yes, minor modification**

**Needed**

We greatly appreciate your feedback.

**Reviewer #3: Figure 1 – Y axis label is incomplete. It should be “Number of hospitalisations”, X axis label is also incomplete - should be Age groups (in years)**

We greatly appreciate your feedback. The file we have uploaded had all suggested label so we will reconfirm them.

**Figures 2 and 3 are redundant because they duplicate the exact data presented in Table 3. Therefore, Fig 2 and 3 should be removed.**

As noted above, the data in figures 2 and 3 recapitulate the age-standardised data presented in Table 3. Table 3 provides raw numbers and percentages while figures 2 and 3 enable readily observable comparisons of these rates across states and remoteness areas. To remove the overlap between the table and figures, the age-standardised rates have been removed from Table 3 and are now only present in the figures.

**Table 3 – footnote “C.I. – Confidence Intervals” is confusing. Why is it stated there? The right-side column head states that “Age-standardised rates per 100,000 population (min-max)”**

References to CI in the footer have been removed.

**Conclusions:**

**-Are the conclusions supported by the data presented?**

**-Are the limitations of analysis clearly described?**

**-Do the authors discuss how these data can be helpful to advance our understanding of the topic under study?**

**-Is public health relevance addressed?**

**Reviewer #1: Although the concluding paragraph suggests that the data supports the aims, in fact, these are just rather broad and ambiguous (motherhood type) statements. This paper will affect health policy - but how? Specifically how is this important in terms of improving the treatment of snakebite.**

**How will this data help with prevention. There is no information on the circumstances of the bite? This is key to preventing it. Is it an occupational hazard, or are they due to people interfering with snakes? How many of the patients were snake handlers? Previous studies have demonstrated that snake handlers make up to 10% of bites in Australia - please discuss, and refer to appropriate research from the Australian snakebite project.**

We thank the reviewer for their comments. We have revised the language of the aims and objectives of the study to provide greater context for the utilisation of the findings from the current study.

**There is no information on pre-hospital treatment, or patients that do not present to hospital. There is no information on deaths. This is not discussed as a limitation.**

The factors noted above have been added to a new paragraph in the limitations sections as noted below.

*The lack of ancillary data to complement the hospital level data in the current investigations means that we were unable to investigate the rate and impact of pre-hospital treatments, such as the recommended pressure immobilisation bandage (21), the rate of complications, such as myotoxicity (ASP-23), thrombotic microangiopathy (ASP-30), and early cardiovascular collapse, as noted by ASP (22), nor the overall fatality rate of venomous bites across the study period. This is a significant limitation of the current hospitalisations data and the findings noted here are thus best utilised in concert with the broader work in the field.*

*It is also noted that the recorded identification of snake species in many of these cases are unlikely to be definitive, instead relying on some combination of clinical presentation, descriptions or photos provided by patients, knowledge of venomous regional species, consultation with state-based Poisons Information Centre, and clinical toxinologists, and the use of snake venom detection kits (SVDKs), when and where available. Despite these limitations, the data here corroborated earlier findings from the Australian Snakebite Project (ASP) which included more definitive snake species identification and reported that brown snake envenoming accounted for 41% of venomous bites in a cohort of 1,548 Australian patients, with tiger snakes and black snakes accounting for 17% and 16% respectively (6).*

**The identification of snakes is not defined - this is a clear limitation because it is well known that many people are bitten by "brown-coloured" snakes, which are not necessarily "brown snakes" - they must discuss this in reference to studies with definite snake identification.**

As noted above the following text has been added to the discussion to highlight how snake type determinations are made in the clinical setting.

*‘It is noted that the recorded identification of snake species in many of these cases are unlikely to be definitive, instead relying on some combination of clinical presentation, descriptions or photos provided by patients, knowledge of venomous regional species, consultation with state-based Poisons Information Centre, and clinical toxinologists, and the use of snake venom detection kits (SVDKs), when and where available.’*

**Probably the largest weakness is that the authors provide a very poor literature review - there are 9 references, many from overseas. There have been numerous studies of snakebite from hospitals, poison centres, most from the Australian Snakebite project, which has not been discussed at all. Comparison of this data to the Australian snakebite project is essential, since it contains much more detailed information on all aspects of snakebite - demographics, circumstances, prehospital information, correct snake identification, clinical effects, outcomes and treatments.**

We thank the reviewer for their comment. We have expanded the references to include a greater variety of Australian references to compare and contrast the current findings, including the noted Australian Snakebite Project.

**The authors do not refer to the recent PAVLOVA study, a much more detailed and finer database linkage of poisoning and envenoming in Australia (for one State).
Cairns R, Noghrehchi F, Raubenheimer JE, Chitty KM, Isbister GK, Chiew AL, Brett J, Dawson AH, Brown JA, Buckley NA. Poisoning and envenomation linkage to evaluate outcomes and clinical variation in Australia (PAVLOVA): a longitudinal data-linkage cohort of acute poisonings, envenomations, and adverse drug reactions in New South Wales, Australia, 2011-2020. Clin Toxicol (Phila). 2024 Oct;62(10):615-624.**

We thank the reviewer for their suggestion and have included reference to the recent PAVLOVA study in the discussion.

**Reviewer #2: -Are the conclusions supported by the data presented? Yes**

**-Are the limitations of analysis clearly described? Yes**

**-Do the authors discuss how these data can be helpful to advance our understanding of the topic under study? Yes**

**-Is public health relevance addressed? Yes**

**Reviewer #3: Well-written**

We appreciate your suggestions, and the discussion has been thoroughly revised, incorporate all possible references.

**Editorial and Data Presentation Modifications?**

**Use this section for editorial suggestions as well as relatively minor modifications of existing data that would enhance clarity. If the only modifications needed are minor and/or editorial, you may wish to recommend “Minor Revision” or “Accept”.**

**Reviewer #1: Nil**

**Reviewer #2: (No Response)**

**Reviewer #3: Both terms, ‘envenoming’ (UK English) and ‘envenomation’ (US English) have been used in the manuscript. It would be appropriate to be consistent with one style.**

References to envenomation have been converted to envenoming to ensure consistency across the manuscript, with the exception of reference titles.

**Summary and General Comments:**

**Use this section to provide overall comments, discuss strengths/weaknesses of the study, novelty, significance, general execution and scholarship. You may also include additional comments for the author, including concerns about dual publication, research ethics, or publication ethics. If requesting major revision, please articulate the new experiments that are needed.**

**Reviewer #1: The major weakness of this paper is that it is presented in isolation to any previous research done in Australia on snakebite. A simple PubMed search of "Australia" and "Snakebite" identifies hundreds of studies very quickly, and a search thru these excluding biomedical research, identifies 10s of papers by other research groups, clinical groups and poison centres on snakebite in Australia.**

The introduction and discussion have been revised to include additional local context to the results. We thank the reviewer for their suggestion. Example text from the introduction is provided below, while changes to the discussion are tracked.

*“Despite being the only continent on which front-fanged venomous snakes outnumber non-venomous species, snakebite rates in Australia remain relatively low due in part to low population density in rural areas, with previous reports indicating that approximately 500-600 Australians are hospitalised each year following contact with venomous snakes (4, 5). Fatal snakebites are rare (2-4 deaths per year on average)(4-6) due to the wide availability of high-quality antivenoms across Australia’s public health care system, with approximately 750 hospitals across the country maintaining a local supply (6-8). When bites do occur, understanding contributing factors such as demographics, geographic locations (e.g. living in rural or remote areas with close proximity to snake habitats), and the behavioural ecology of the snakes involved, can help refine our understanding of the impact of snakebite across the country. Equally, insufficient knowledge and lack of identification of venomous snakes, inappropriate first aid measures, and delays in reaching hospital or access to healthcare may exacerbate the consequences of snakebite.*

*In the early 1990s, it was estimated that approximately 3,000 people were bitten by snakes each year in Australia, with 200 of these individuals, at a minimum, receiving antivenom (9). While the accuracy of these estimates was unclear, it had been established that venomous snakebites killed on average 1 to 4 people each year across the country (9, 10). More recent investigations have indicated that the number of people hospitalised in Australia following contact with venomous snakes is closer to 450-600 people each year (5, 11, 12). Studies have reported that between 25-55% of patients with suspected bites from venomous snakes exhibit signs of clinical envenoming (5, 6), with the lower rates likely including dry bites (snakebites where venom was not delivered/injected into the bitten individual), and “stick” bites (injuries caused by sharp sticks or twigs often in combination with the sighting of a nearby snake), in addition to clinically meaningful snakebites.”*

**Reviewer #2: Overall, Australia has no native venomous lizards, and the details of lizards' injuries have made this manuscript less valuable, unless the lizard's injuries can be described in detail. No information about actual lizard injuries has been described. If possible, highly recommend excluding the lizard's details and presenting the data only on snakebites and envenomation. Especially under the results, presenting envenomation, antivenom treatments make no sense after including cases of non-venomous lizards.**

We agree with the sentiment of the comments made by Reviewer 2 and have added the following text to the methods data definition section to clarify this.

*“The X20 ICD code is an international code which accounts for countries where contact with venomous lizards is a medically significant event. Australia does not have medically significant venomous lizards and so this code has been shortened to X20 Contact with venomous snakes throughout the remainder of this article.”*

**Under discussion: Present incidence, envenomation, antivenom treatment for snakebites, state-wise, published in previous literature and compared with the newly extracted data.**

The discussion has been substantially revised to include a broader discussion of the findings noted above in the context of the broader literature.

**Introduction: Include details of the incidence of snakebite and the envenomation in Australia, expressed per state.**

Details of the incidence of snakebite and envenoming in Australia have been added to the introduction as noted above. State-based comparisons have been included in the discussion.

**Second paragraph, 3rd line: India is part of South Asia, not Southeast Asia.**

This has been amended.

**Since most of the Australian territories are vast, it is important to present a map of the locations of the hospitals, indicating where the hot spots are. This is important to make the data more sense in policy making and implementing preventive strategies.**

We are unable to provide details at this level based on our current ethics approvals but agree that this data would be beneficial for policy makers.

**Discussion, paragraph 1: Why are these data not presented under the results, and only in the discussion?**

All data presented in the discussion is first presented in the results section.

**Data present in many places in results and discussion: Please add the number where you have presented the data as a %.**

**Figure 1: Add % on the top of each bar.**

**S4: Delete the repeated word “due to” in the caption.**

**Many places: Unnecessary bold and italic formatting of: (Tables and Figures)**

**Reviewer #3: This manuscript describes the trends of hospitalisation due to snake and lizard -related injuries across Australia during 2002 to 2020. It concludes that despite population growth, urban expansion, and climate change, snakebite hospitalization rates in Australia remained stable during the above period. The manuscript includes some important data, for the global literature. However, there are some issues that need addressing.**

We thank the reviewer for their comments and have attempted to address the concerns raised as noted above.

**PLOS authors have the option to publish the peer review history of their article (**[**what does this mean?**](https://track.editorialmanager.com/CL0/https:%2F%2Fjournals.plos.org%2Fplosntds%2Fs%2Feditorial-and-peer-review-process%23loc-peer-review-history/1/010f01973fbe3cba-317f6d1f-7863-4f65-b6f9-65a5a982f6a7-000000/EvNT6zmHj5dxG_yiy2ntUA0UzFF6hWeuQTR6q3rkRBU=214)**). If published, this will include your full peer review and any attached files.**

**If you choose “no”, your identity will remain anonymous but your review may still be made public.**

**Do you want your identity to be public for this peer review? For information about this choice, including consent withdrawal, please see our** [**Privacy Policy**](https://track.editorialmanager.com/CL0/https:%2F%2Fwww.plos.org%2Fprivacy-policy/1/010f01973fbe3cba-317f6d1f-7863-4f65-b6f9-65a5a982f6a7-000000/lyFwENKS0WTwXEvpmi0An0h1g6LYZx2b-OhB6lZcU0o=214)**.**

**Reviewer #1: No**

**Reviewer #2: Yes: Kalana Maduwage**

**Reviewer #3: No**
